# Concise review: Nanoparticles and cellular carriers-allies in cancer imaging and cellular gene therapy?

**DOI:** 10.1002/stem.473

**Published:** 2010-07-13

**Authors:** Catherine Tang, Pamela J Russell, Rosetta Martiniello-Wilks, John E J Rasko, Aparajita Khatri

**Affiliations:** a Oncology Research Centre, Prince of Wales HospitalRandwick, Sydney, NSW, Australia; b Faculty of Medicine, University of New South WalesKensington, NSW, Australia; c Gene and Stem Cell Therapy Program, Centenary Institute, University of SydneyNSW, Australia; d Cell and Molecular Therapies, Royal Prince Alfred HospitalNSW, Australia

**Keywords:** Stem cell tracking and imaging, Magnetic nanoparticles, Mesenchymal stem cells, Cancer, Nanotechnology, Gene therapy, SPION

## Abstract

Ineffective treatment and poor patient management continue to plague the arena of clinical oncology. The crucial issues include inadequate treatment efficacy due to ineffective targeting of cancer deposits, systemic toxicities, suboptimal cancer detection and disease monitoring. This has led to the quest for clinically relevant, innovative multifaceted solutions such as development of targeted and traceable therapies. Mesenchymal stem cells (MSCs) have the intrinsic ability to “home” to growing tumors and are hypoimmunogenic. Therefore, these can be used as (a) “Trojan Horses” to deliver gene therapy directly into the tumors and (b) carriers of nanoparticles to allow cell tracking and simultaneous cancer detection. The camouflage of MSC carriers can potentially tackle the issues of safety, vector, and/or transgene immunogenicity as well as nanoparticle clearance and toxicity. The versatility of the nanotechnology platform could allow cellular tracking using single or multimodal imaging modalities. Toward that end, noninvasive magnetic resonance imaging (MRI) is fast becoming a clinical favorite, though there is scope for improvement in its accuracy and sensitivity. In that, use of superparamagnetic iron-oxide nanoparticles (SPION) as MRI contrast enhancers may be the best option for tracking therapeutic MSC. The prospects and consequences of synergistic approaches using MSC carriers, gene therapy, and SPION in developing cancer diagnostics and therapeutics are discussed. STEM CELLS 2010; 28:1686–1702.

## CURRENT ISSUES IN CANCER IMAGING AND THERAPY

Approximately 25 million people live with cancer [1] and ∼13% of all deaths are attributed to this disease [2] worldwide. As specific molecular technologies improve, cancer is increasingly recognized as a highly heterogeneous disease. Despite improvements in anticancer therapies, the lack of tumor-specificity results in significant treatment-associated morbidity, ultimately limiting efficacy due to dosage limitations. Research priorities must now seek to refine the specificity and accuracy of cancer detection and treatment as well as develop strategies that target a wider repertoire of cancer cells. An important aim should be to achieve optimal patient management and improved quality of life through early detection of cancer and metastases, improved treatment delivery, and monitoring of outcomes through accurate and sensitive imaging techniques. Although magnetic resonance imaging (MRI) and computed tomography (CT) are currently integral to patient assessment and management, lesions <1 cm are still difficult to detect owing to the subjective nature of interpretation that may lead to inaccurate assessment [3,4].

Recent developments in real-time in vivo imaging technologies using image contrast enhancers offer tangible options to better guide treatment delivery and monitor outcome. Furthermore, improved treatment specificity may be achieved through gene therapy-based approaches. Using viral and nonviral vectors, genetic material can be specifically targeted to cancer cells, for example, to compensate for mutations in tumor suppressor genes, to potentiate anticancer immune responses, or to cause oncolysis [5]. However, obstacles to effective delivery of both contrast agents and gene vectors remain. Immune and reticuloendothelial sequestration or nonspecific vector uptake by nontarget organs dramatically reduces treatment efficacy. No single agent has offered a solution, but recent developments in cancer targeting using stem cell (SC) carriers and nanotechnology have led to innovative possibilities. We discuss the prospects of using SCs as gene therapy carriers and review strategies combining these with nanocarriers to facilitate monitoring and therapy.

## SCs AS CARRIERS OF CANCER THERAPY

The ability of SCs to migrate to pathological sites including wounds, ischemia, and cancer (including micrometastases) [6–13] underpins their development as carriers of therapy, thus, providing an exciting paradigm for targeted cancer therapeutics. The importance of the microenvironment in tumorigenesis was first recognized in Paget's seminal (1889) “seed and soil” hypothesis [14]. Stroma provides the architectural framework for tumor development while facilitating molecular crosstalk via cytokines and growth factors to promote cellular turnover and angiogenesis. Thus, tumorigenesis closely resembles wound healing, leading to description of tumors as “wounds that do not heal” [15]. Further, extracellular matrix (ECM) remodeling is mediated by SC and tumor cells [16–18].

**Figure 1:**
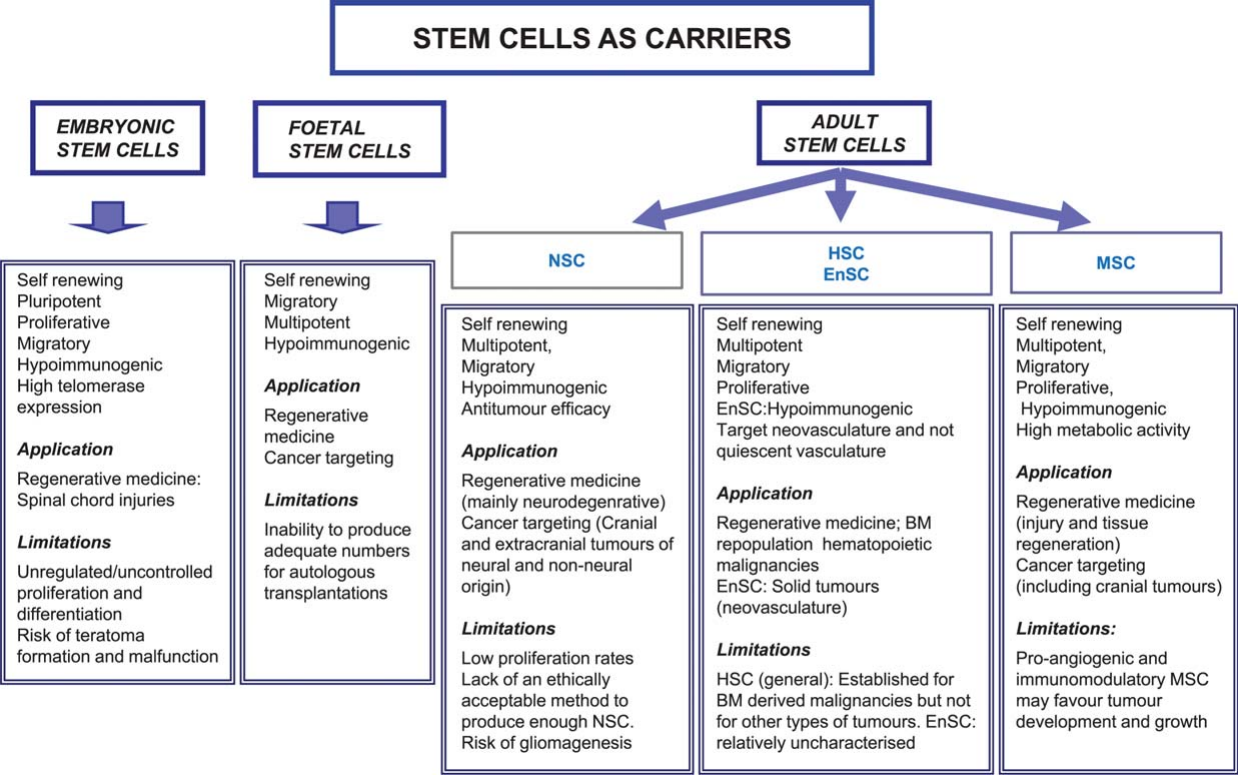
A schematic summarizing the properties, applications, and limitations of different stem cells for the treatment of biomedical conditions including cancer. Abbreviations: BM, bone marrow; EnSC, Endothelial Stem Cells; HSC, hematopoietic stem cell; MSC, mesenchymal stem cell; NSC, neuronal stem cell.

SCs from different sources have been explored for biomedical applications: embryonic SC; fetal multipotent SC; induced pluripotent SC; adult multipotent SC comprising neuronal SC (NSC), hematopoietic SC (HSC), and mesenchymal SC (MSC) (reviewed in [11]; Fig. 1 summarizes their properties, potential applications, and drawbacks). Overall, by virtue of their lineage plasticity and tumor tropism, adult SCs display the best attributes for targeting cancer. Both HSC and NSC have been explored with variable success, however, their application is limited either due to issues with production or inadequate characterization (Fig. 1; reviewed in [19–25]). MSCs are currently under intense investigation as potential clinical therapeutic carriers due to their high lineage plasticity [26] and minimal ethical concerns associated with their isolation and use [11]. This review will focus on the potential of MSC as cellular carriers in oncology.

## MSCs AND CANCER

MSCs are multipotent stromal cells with the ability to self-renew, differentiate into cells of diverse lineage [27], and migrate to sites of pathology [28]. First isolated as an adherent mononuclear cell fraction of bone marrow (BM) [29], MSCs are present virtually in all postnatal tissues [30]. The following MSCs properties make them ideal therapeutic cellular carriers (Table 1): ease of isolation and expansion in vitro; ease of ex vivo genetic modification; autologous transplantation in patients (overcome issues of host immune responses); and finally, hypoimmunogenicity (suitable for allogeneic transplantations). Indeed, approval of ∼107 clinical trials employing MSCs for regenerative medicine, stroke, and myocardial infarction (http://clinicaltrials.gov/ct2/results?term=Mesenchymal+stem+cells&show_flds=Y) [47] suggests the clinical feasibility of their use for cancer targeting.

**Table 1:** Properties of MSCs relevant to applications in cancer imaging and therapy

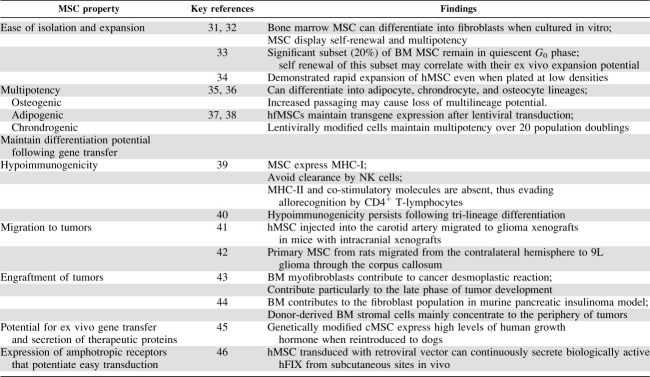

Abbreviations: BM, bone marrow; cMSC, canine MSC; hFIX, human factor; hfMSC, human fibroblastic MSC; hMSC, Human MSC 8; MSC, mesenchymal stem cells.

## MSCs AND TUMOR TROPISM

MSCs show preferential migration toward sites of inflammation, injury, and cancer [6]. Typically, these are attracted to lesions where they engraft into the stroma and persist: in xenograft experiments, 40% of intratumoral fibroblasts in pancreatic lesions in mice were of BM origin [44]. Although, distributed throughout the tumor mass, both this and subsequent studies have shown a greater concentration of BM-derived cells toward the tumor periphery, indicative of the role of MSCs in the later stages of stromal induction as regulators of desmoplastic reactions [48]. Thus far, the tropism of MSCs for gliomas [42,49], pulmonary metastases [50–52], breast cancer metastases [53] ovarian carcinoma [54], and melanoma [55] has been demonstrated in several animal models.

Although not completely understood, MSCs “homing” to cancer may involve recruitment of resident fibroblasts and circulating MSCs into the tumor microenvironment through the release of growth factors and chemokines, where they proliferate and subsequently differentiate into tumor stroma forming fibrocytes, myofibroblasts, and neovascular pericytes [48]. Chemokine-receptor pairs including stromal-derived growth factor SDF-1/CXC chemokine Receptor-4 (CXCR4) [56], monocyte chemotactic protein-1/chemokine (C-C motif) receptor 2 [53], hepatocyte growth factor/c-met [57], and Vascular Endothelial Growth Factor (VEGF)/VEGF receptor [58] together with ECM proteins have been implicated [59] (reviewed in [60]). A clear understanding of these processes is crucial to improve MSCs “homing” to tumors in vivo. Characteristics unique to their migratory phenotype including the chemokine receptor status and triggering events such as cytokine release and matrix metalloproteinase (MMP) production at tumor site need to be identified to determine the optimal biological window for therapeutic MSCs targeting of tumors. For example, postresection production of cytokines that recruit MSCs to gliomas [61] could provide a window to target gliomas with therapeutic MSCs to remove residual disease. Further, to achieve optimal tumor targeting, specific identification of nonquiescent SC populations, which can migrate, target, and integrate into tumor tissue, is essential. This would require an assessment of relevant receptors on these cells and their responses to biological triggers using molecular imaging and appropriate ex vivo or in vitro three-dimensional models [62].

## MSCs AND CANCER GENE THERAPY

Overall, the recognition that MSCs “home” toward tumors while evading immune clearance has led to extensive research into their use for cancer-specific gene delivery [11,48,50,55]. A primary consideration for such applications is to ensure their in situ efficacy and survival with the retention of their fundamental properties of migration, differentiation, and hypoimmunogenicity, after modification.

Cancer gene therapy delivered using MSC has been based on suicide-, apoptosis-, anti-angiogenesis-, immuno-stimulatory genes, or oncolytic viral vectors (reviewed in [63]) primarily, using the viral vectors. The use of MSCs as carriers for these vectors [5] can address the drawbacks associated with their direct use including: safety (e.g., insertional mutagenesis when integrating viral vectors [retroviruses] are used) [64]; inadequate tumor targeting; inefficient gene delivery resulting from vector and/or transgene immunogenicity; limited availability of virus-specific “receptors” on cancer cells or inefficient transduction of nondividing cells. Furthermore, ex vivo MSCs manipulation maximize transduction efficiency by allowing for the selection of cells carrying the desired gene before in vivo delivery.

### Viral Vectors and MSC

Transduction of MSCs by integrating retroviral vectors is efficient, but their random genomic integration can lead to unwanted transformation, significantly increasing the risk of secondary malignancies. Despite continuing efforts toward the assessment and accurate mapping of safe insertion sites, currently, the risks may outweigh the advantages. Hence, nonintegrating vectors, such as adenoviruses (Ad), are appealing and are the most widely explored for cancer gene therapy. Ad can be grown to high titer (∼10^12^ virus particles per milliliter), yield high gene expression and importantly, transduce dividing and nondividing cells [64]. However, systemically administered Ad can be rapidly cleared by the immune system and hepatic Kupffer cells [64] and inactivated by Ad-neutralizing antibodies in humans [65]. This substantially compromises the efficiency of Ad gene delivery [66–69]. Because of their hypoimmunogenicity, MSCs may act as a “Trojan Horse” for the delivering Ad-mediated gene therapy directly into tumor lesions. This concept has generated significant interest and is the focus of the following section.

### MSCs as Hypoimmunogenic Cellular Vehicles for Adenoviral Vectors

MSCs express major histocompatibility class (MHC)-I antigens, thereby avoiding clearance by natural killer cells, whereas the absence of MHC-II and costimulatory molecules permit immune evasion from CD4^+^ T-lymphocytes [39]. In vitro studies have demonstrated that MSCs do not cause the proliferation of allogeneic T-cells following interferon (IFN)-γ stimulation [70] and this hypoimmunogenicity persists even after tri-lineage differentiation [40]. Studies in several animal models (rodents, dogs, pigs) have shown that allogeneic-mismatched MSCs can engraft in vivo [71]. Importantly, recent studies have shown that MSC-Ad produce therapeutic transgenes even in the presence of physiological concentrations of sera that would otherwise neutralize adenovirus alone in vitro [52]. Thus, the dual benefits of MSCs homing and their potential for allogeneic transplantation without extensive immunosuppression can be exploited to increase Ad-gene delivery specifically to tumor sites.

### Efficacy of MSCs Carrying Therapeutic Genes Against Cancer

High metabolic activity of MSC permits high-level transgene expression [72]. The use of MSC carriers to deliver Ad-vectors expressing therapeutic genes has been assessed in several preclinical models of cancer (Table 2). Specific delivery of cytokine transgenes to tumor sites has been attempted to mitigate the toxicities associated with systemic administration of the corresponding recombinant proteins. Unlike systemically administered IFN-β, systemically delivered MSC-expressing IFN-β suppressed tumor growth and prolonged survival in a lung melanoma model [50]. The antitumor effects were attributed to the local production of IFN-β within the tumor, thus, highlighting the importance of MSC engraftment for cancer-targeted delivery [34]. Similar benefits have been achieved using MSC-expressing interleukin-2 [42], fractalkine [74], and tumor necrosis factor-related apoptosis inducing ligand [52] against intracranial glioma, lung metastases, and lung carcinoma, respectively. The localized production of cytotoxic drug metabolites was also achieved using MSC-expressing cytosine-deaminase; local conversion of the prodrug, 5-fluorocytosine to 5-fluorouracil, resulted in inhibition of growth of colorectal cancer [76] and melanoma [77] xenografts.

**Table 2:** Efficacy of adenovirus-transduced MSCs in preclinical tumor models

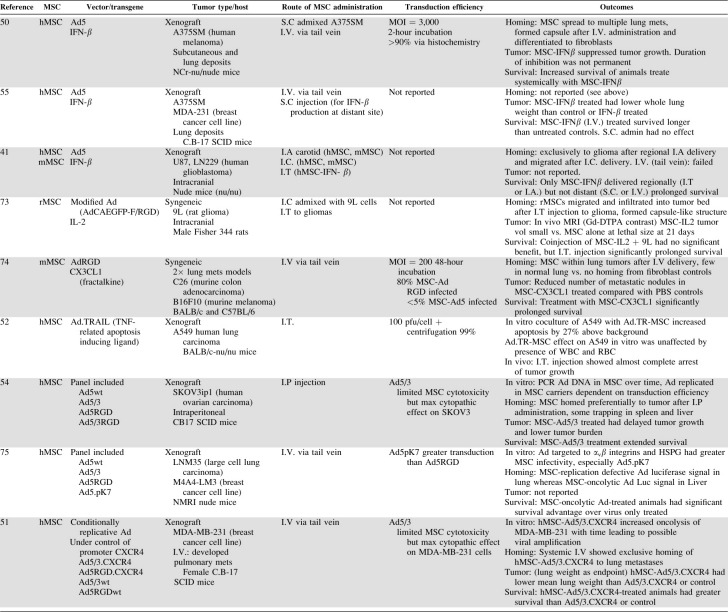

Ad5/3, Ad5 with chimeric fiber(Ad5+Ad3); Ad5, adenovirus serotype 5; Ad5.pK7, Ad5 with fiber containing polylysine (7 residues); Ad5RGD, Ad5 with integrin binding RGD motif in its fiber; Ad5wt, Ad5 wild type; AxFAEGFP-F/RGD, adenoviral vector carrying humanized variant of *Aequoria victoria* green fluorescent protein with RGD-mutated fiber under control of a CA promoter; CX3CL1, C-X3-C-motif ligand 1 (Fractalkine); CXCR4, cxc chemokine receptor 4 (Fusin); hMSC, human MSC; I.A., intraarterial; I.C., intracranial injection; IFN-β, Interferon beta; IL-2, interleukin-2; I.P., intraperitoneal injection; I.T., intratumoral injection; I.V., intravenous injection; mMSC, murine MSC; MOI, multiplicity of infection; MSC, mesenchymal stem cells; PBS, phosphate buffered saline; S.C., subcutaneous.

MSCs have also been used to carry and support the replication of oncolytic viruses, which infect tumor cells when released into the tumor mass. This strategy relies on the optimal balance between minimizing cytotoxicity to the cellular carriers and maximizing cytopathic effects on cancer cells. Thus, systemically delivered MSC-bearing oncolytic viruses have been successful against lung metastases [51] and orthotopic breast and lung tumors [75] displaying cytopathic effects against cancer cells with minimal toxicity to the MSC themselves. Similarly, extended host survival and delayed tumor growth was seen following intraperitoneal delivery of MSC-bearing oncolytic Ad against ovarian cancer [54]. In comparison, the same doses of virus injected systemically showed only liver accumulation [75], validating the MSC cell-carrier approach to more efficiently target cancer. However, the low transduction efficiency of MSC with Ad vectors due to the low expression of Ad-receptor Coxsackie-adenovirus receptor (CAR) is a limiting factor [78]. This could not be improved by increasing the multiplicity of infection or time of exposure to the Ad [79]. However, alteration of the Ad tropism with genetic modification of Ad-fiber-knob to contain poly-L-lysine or addition of the Ad35 fiber improved MSC transduction by16- to 460-fold [80]. Cumulatively, these studies indicate the superiority of Ad-modified MSC over the use of Ad alone, although, their therapeutic success is conditional on the efficiency at which MSC are transduced and their subsequent engraftment and persistence within the tumors.

### Limitations of MSC Carriers for Cancer Gene Therapy

Despite tremendous interest in MSC, the unpredictability of their in vivo biological properties such as migration and potential for contribution to the neoplastic phenotype poses a serious obstacle. Some studies have raised concerns that proangiogenic and immunomodulatory properties of MSC may potentiate growth and metastatic capacity of epithelial cancer cells particularly when MSCs are mixed with the cancer cells prior to implantation [81]. Others have shown no apparent effect of exogenous MSC on tumor progression with proven MSC migration but lack of proliferation and differentiation [82,83]. Thus, exhaustive investigative studies are essential prior to any clinical application [63], for example, an assessment of the time required to generate pathology free cells, genetic modification, expansion and phenotypic and genotypic characterization, and then certification for human use needs to be established. The investment in time and resources to produce clinical grade MSC showing acceptable levels of genetic modification and expansion to a therapeutic dose for use in patients is considerable. A timely completion of such characterizations is particularly challenging when dealing with primary cells prior to transplantation. Furthermore, MSC from different sources show different properties [84,85]. For example, MSC from BM proliferate less efficiently than those from umbilical chord or adipose tissue. Some of these constraints can be addressed by development of immortalized, clonal MSC lines that after exhaustive characterization, especially, with respect to their tumorigenicity (e.g., based on type of immortalization gene, its insertion site), may be optimal for clinical use [63].

Additionally, variations in persistence, survival, and interactions of MSC in the tumor microenvironment can affect the duration and level of gene expression at the tumor site [63]. Chemotherapy or the immunogenicity of the transgene and/or vector may impact on MSC survival in situ. Overall, adequately long systemic survival of these carriers is mandated to ensure therapeutic efficacy against cancer. This could in part be supported by the production of immunosuppressive chemokines or cytokines, such as VEGF, Interleukin 10 (IL10), or immunosuppression of T effector-, antigen-presenting- or regulatory T-cells. A better understanding of MSC biology can be exploited to prolong their systemic survival, for example, recognition that CD47 marker expression can prevent SC phagocytosis by macrophages [86,87]. Ultimately, all new approaches must be assessed using human data to evaluate safety and efficacy. Toward that end, a rapid translation of the findings will be greatly facilitated by monitoring SC survival and behavior in vivo through use of longitudinal, noninvasive imaging technology [4,82,88–91].

## MSCs AND IMAGING

To translate MSCs benefits to the clinic, their accurate detection and localization in real time using clinically relevant imaging techniques is essential. An ideal imaging modality should be noninvasive, sensitive, and provide objective information on cell survival, function, and location. In context of cancer, MRI, CT, positron emission tomography (PET), and single photon emission computed tomography (SPECT) are the most explored (specific features of different imaging modalities are reviewed in [3,91–95]. Overall, while nuclear imaging by PET or SPECT leads to greater sensitivity (>5 × 10^3^ cells; [96]), these are primarily limited by lack of anatomical context [97]. MRI provides accurate anatomical detail but does not yield information about cell viability and show poor sensitivity (>10^5^ cells; [98]). Although, none of these modalities is ideal, MRI is the most preferred for cellular tracking (comprehensively reviewed in [95,99–102]). Through detection of proton relaxations in the presence of magnetic field (1.5 Tesla [T]–3 T for clinical imaging), it provides tomographic images with excellent soft tissue contrast and can locate the cells of interest in context of the surrounding milieu (edema or inflammation) [103–105] without the use of harmful ionizing radiations (as with CT, PET, SPECT). In addition, MRI offers a greater tracking window in comparison to PET and SPECT that are limited by the decay of short-lived radioactive isotopes.

During MRI, the intrinsic tissue contrast is affected by local microenvironment including magnetic inhomogeneities of the contrast agents, usually measured as changes in two relaxation time constants *T*1 (brightening) and *T*2 (darkening) times [106]. In this context, nanotechnology-based contrast agents have rapidly come to the forefront to improve SC detection in situ. In the following sections, after a brief introduction of the nanotechnology platform as it applies to cancer targeting its potential synergistic applications involving the MSCs carriers are discussed.

### Nanotechnology and Cancer: Potential for Synergies with Cellular Carriers for Targeting Cancer

Initiated by the discovery that particles of ∼50–100 nm “passively” accumulate in cancer deposits, nanotechnology has fast emerged as a tool for imaging and/or delivery of therapies in oncology. This passive uptake occurs via an “enhanced permeability and retention effect” where inherently leaky tumor vasculature coupled with poor intratumoral lymphatic drainage allows extravasation and entrapment of the nanoparticles [107,108]. Further, nanoparticle surfaces can be modified with cancer-specific antibodies or peptides for the “active” targeting of tumor cells [109]. Thus, nanotechnology platforms offer flexibility and versatility. Not only can nanoparticles deliver targeted therapeutic payloads (drugs or genes), their intrinsic components can simultaneously serve as enhancers for imaging [110]. For optimal targeting and efficacy in vivo, these particles should be biocompatible (based on their size, shape, surface coatings, and chemical or immunotoxicity [111,112]), easily targeted (through surface interactions with cancer targeting antibodies, peptides or ligands), and easily tracked by virtue of their composition to allow clinical imaging [110].

Of the ever expanding catalogue of nanoparticles including polymers, dendrimers, liposomes, carbon nanotubes, nanoshells, and magnetic nanoparticles [110], several have gained Food and Drugs (FDA) approval for cancer therapeutics (Doxil, DaunoXome) and imaging (Resovist) [107,110,113]. However, their first clinical application may be as imaging agents [114–116] and in that the best developed are superparamagnetic iron-oxide nanoparticles (SPION).

SPION comprise a crystalline iron-oxide core coated with biocompatible materials such as, dextran, starch, or polyol derivatives, that confer stability in vivo and can be conjugated with cancer-targeting ligands or gene-vectors for active targeting. These display magnetism only under the influence of an external magnetic field [117], which also avoids self-aggregation. Importantly, SPION are biocompatible and are eliminated through the body's normal iron metabolism. SPION have been studied for cancer therapy (hyperthermia), magnetic field-assisted targeting, and as contrast enhancers for MRI and targeted molecular imaging [118,119]. The promise shown in such studies have initiated clinical evaluation of SPION for the detection and management of liver metastases with enhanced sensitivity of up to 95% [120] to nodal metastases in both head and neck [121] and genitourinary cancers [122,123]. Although these studies have shown SPION usage to be safe [124], some issues associated with their use need to be addressed.

#### Issues with Use of Iron-Oxide-Based Nanoparticles.

Major limitations of SPION beyond MRI of the Reticuloendothelial system (RES), include their uptake by phagocytic cells leading to their rapid clearance from the blood [125], in vivo toxicity resulting from the coating materials, and surface chemistry together with unwanted cellular or tissue distribution [118,126]. SPION can also cross the blood-brain barrier and accumulate in the liver (80%–90%), spleen (5%–8%), and BM (1%–2%) [127]. Their ability to agglomerate in the presence of a magnetic field can cause embolization [126,128]. Excessive iron-oxide could also lead to an imbalance in its homeostasis and may lead to toxicity [129]. Therefore, toxicity of any new formulations of SPIONs has to be established and would require extensive characterization terms in terms of SPION composition, coatings, size, and dosing regimens in vivo. Thus, the use of the nanoparticles under the “camouflage” of MSCs may resolve some of these issues. Particularly for cellular tracking, the delivery of nanoparticle-labeled MSCs directly into tumor deposits will not only allow the tracking of the labeled cells but also the targeted tumor deposits.

### Magnetic Nanoparticles and Tracking of SCs In Vivo

Several paramagnetic and magnetic nanoparticles have been evaluated for labeling SCs to enhance their tracking by MRI. Paramagnetic gadolinium (Gd)- and Mn-based nanoparticles lead to image brightening (*T*1-based) while those based on SPIONS (50–200 nm), ultraSPION (∼35 nm), and micron-sized (MPION) lead to image darkening (*T*2 and *T*2*-based) [114,130]. Of these, only SPION are approved for clinical imaging and are the general focus of this review.

#### SPION and MRI of SCs.

**Figure 2:**
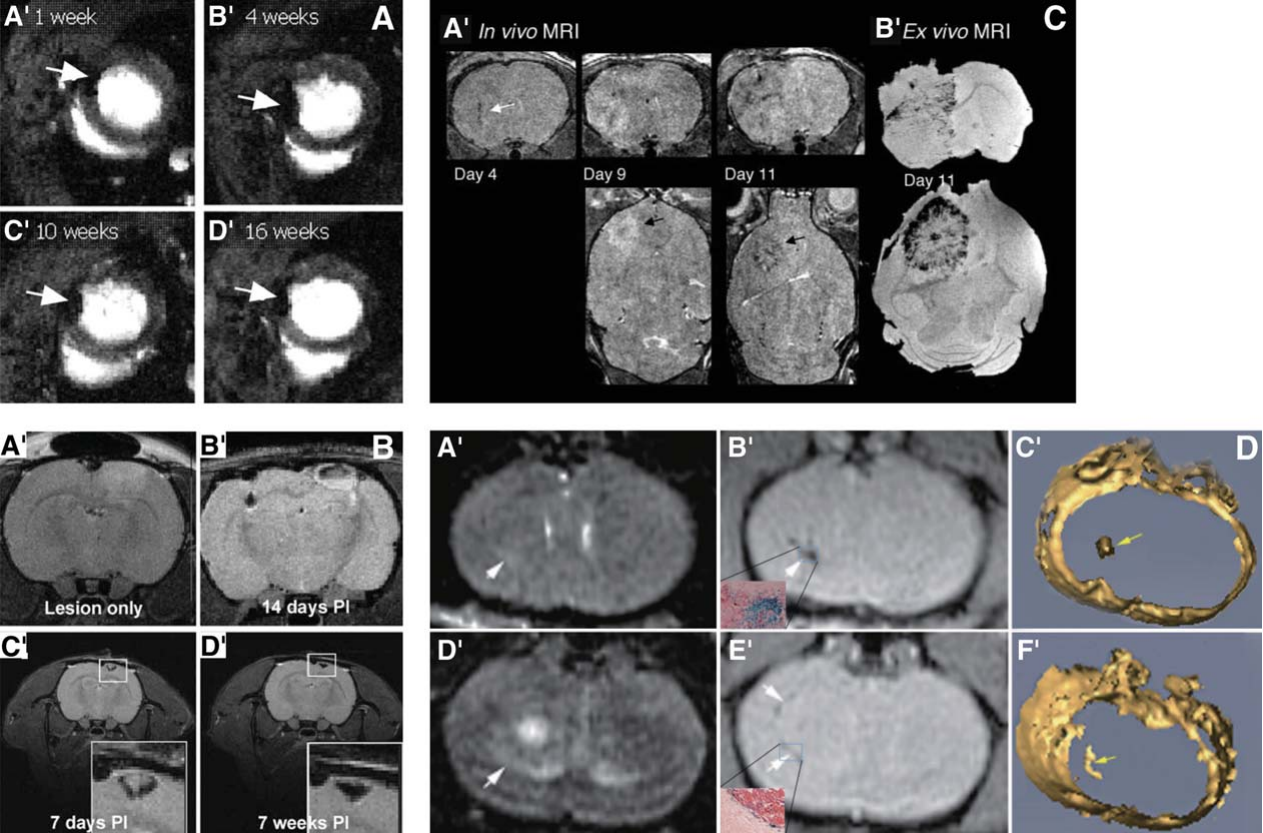
MRI of superparamagnetic iron-oxide nanoparticles (SPION)-labeled stem cells showing their persistence, migration, and tumor homing in vivo. **(A)**: Demonstrates long-term mesenchymal stem cell (MSC) traceability using SPION. Rat MSCs labeled with iron particles injected into the infracted heart could be detected as hypointense regions from 1 week and detected for up to 16 weeks. Volume of the signal void reduced to lesser extent in severely infarcted hearts in comparison with milder infarcts. ©AlphaMedPress, April 20th, 2006; Reprinted from [134], with permission from Wiley-Liss, Inc. a subsidiary of John Wiley & Sons, Inc. **(B)**: Migration of MSCs to site of pathology: Endoderm (SPION)-labeled rat MSCs migrate toward the lesion of brain (A′) could be detected by MR up to 7 weeks after implantation (C′ and D′) in the contralateral hemisphere MSCs (B′). Reprinted from [7], with permission from Macmillan Publishers Ltd., © 2007 Nature Publishing Group. **(C)**: Tumor homing and engraftment by Sca-1 positive bone marrow (BM) cells (target the tumor vasculature) by MRI: Serial MRI in tumor bearing mice that received magnetically labeled Sca-1^+^ BM cells. (A′): Three-dimensional (3D) RARE images show dark regions developing within and around tumors due to incorporation of labeled cells into the vasculature and parenchyma of tumor, images are acquired on day 4, 9, and 11. By day 11, a dark rim appear on the tumor periphery. (B′): Corresponding ex vivo gradient images of the same mouse on day 11. MR evidence of labeled cell incorporation demonstrates that neovascularization occurs primarily at the tumor periphery in the later stages of tumor development. Reproduced from [135], with permission from (This research was originally published in *Blood*) © 2007 The American Society of Hematology. **(D)**: Tumor homing by SPION-labeled MSCs: The pattern of MSCs distribution, their incorporation and migration could be tracked using 1.5-T MR imaging following i.v. injection of SPION/green fluorescent protein-labeled cells. MSCs distribution throughout the tumor on day 7 (B′) was shown by a well-defined dark hypointense region. After 14 days, most MSCs were found at the tumor border (hypointense region in [D′]), (C′, F′). 3D reconstructions show the SPION-labeled MSCs as yellow structures indicated by the yellow arrows. This study demonstrates that systemically transplanted MSCs migrated toward glioma with high specificity in a temporal–spatial pattern. Reproduced from [49], with permission from ©1944-2009 by the American Association of Neurosurgeons. Abbreviations: MRI. magnetic resonance imaging.

SPION display greater magnetic susceptibility in comparison to conventional Gd and engender significant signal loss to delineate areas of interest. Thus, SPION-labeled cells display a “blooming” artifact' that extends beyond the size of particles making the cells more visible for detection. Clinically, about 1–30 pg Fe per cell is adequate for detection of labeled cells by MRI without alterations in the proliferation, migration, differentiation, reactive oxygen species formation, and apoptosis rates [118,131]. Hence, with the increasing use of MSCs for therapy of tissue injury [132,133], MRI tracking protocols have gained prominence generating crucial information about their migration and survival. MRI signals from intramyocardial implanted SPION-labeled MSC could be detected for up to 16 weeks (Fig. 2A) [132–134] and specific migration of intravenously given SPION-labeled MSCs to the infarct area and not to the healthy surrounding viable myocardium was shown [136]. Further, through MRI of SPION-labeled porcine MSC, improved survival in the infarct zone than in healthy myocardium was shown [137]. Similarly, in models of brain injury and stroke, injury-specific migration of SPION-labeled MSCs (injected contralateral to the area of injury or infused intravenously) could be tracked by MRI (Fig. 2B) [8,28,138,139]. Thus, MRI-based demonstration of retention of injury-specific migration and improved survival of labeled MSCs at the site of injury suggests the feasibility of this approach in clinical oncology.

#### Targeting Cancer and Cellular MRI.

In cancer, to date, cellular MRI has primarily been explored in glioma models with most studies employing EnSC or NSC. MRI of SPION-labeled endothelial progenitor cells demonstrated their tumor tropic migration and differentiation into neovasculature within intracranial glioma (Fig. 2C) [135,140]. Given their neuronal bias, NSC carriers are the most explored for targeting glioma, (NSC literature for reference: [9,35,25,63,99,141–143]). Indeed, through MRI of magnetically labeled NSC, their seeding, migration, homing to invading tumor cells has been evaluated successfully clearly indicating the promise of such combinational approaches [9,144–151] for tracking cellular carriers to cancer lesions. In that, MSCs have generated recent interest as unlike NSCs, these are readily expandable with minimal ethical issues. MRI of intravenously infused SPION-labeled MSCs demonstrated specific migration toward glioma MRI [49] in a temporal-spatial pattern showing initial distribution throughout the tumor with subsequent concentration at the periphery (Fig. 2D) [49]. MRI at 1.5 T could detect the signal for over a week with ensuing decay after 14 days, but could be improved by MRI at higher magnetic field strengths. As glioma has a diffuse distribution and spreads beyond the original site [152], the ability of MSCs to home toward metastatic glioma highlights their potential to “track” the migration of cancer. The translation of such noninvasive imaging techniques toward a broader repertoire of cancers is now being explored.

### Approaches to Improve MRI Sensitivity and Duration

Magnetic-nanoparticle-labeled cells face limitations typical of exogenously labeled cells such as the dilution of signal with cell division limits the duration of MRI tracking; attenuation of signal down to 42% of the original after 8 weeks was observed [153]. Further, asymmetric sequestration of label during cell division may compromise detection accuracy [154]. The accuracy of MRI data is also compromised by the inability to distinguish viable and nonviable cells and the generation of false signals from dead cells or those engulfed by macrophages [155]. This has initiated interactive research to improve cellular MRI sensitivity and accuracy; a discussion of some of the approaches follows.

Efficient labeling of MSCs can improve detection by magnifying MRI signals [102,156]. Given that spontaneous SPION uptake is minimal in virtually all cell types apart from those of the RES [157], attempts have been made to increase iron loading into cells through SPION derivatization with peptides [158], dendrimer coatings [159], combination with transfection agents (TAs) [131,160–162], and electroporation [163] with variable success (Table 3). Currently, the most widely accepted protocols involve combining SPION with TAs such as poly-L-lysine and protamine-sulfate, achieving high labeling efficiency approaching 100% [161,162]. However, the narrow therapeutic index for titration of these TAs raises the chance of cytotoxicity, changes in the gene expression or their migratory ability [168,180,181]. Furthermore, while SPION-TA-transduced MSCs display unaltered adipogenic and osteogenic differentiation, there is continued debate regarding their deleterious effects on chondrogenesis [131,170]. Thus, a special emphasis on new ways to maximize iron internalization in MSCs while limiting toxicity and impact on normal MSCs properties is needed (Table 3).

**Table 3:** Potential strategies to increase MSC uptake of magnetic nanoparticles

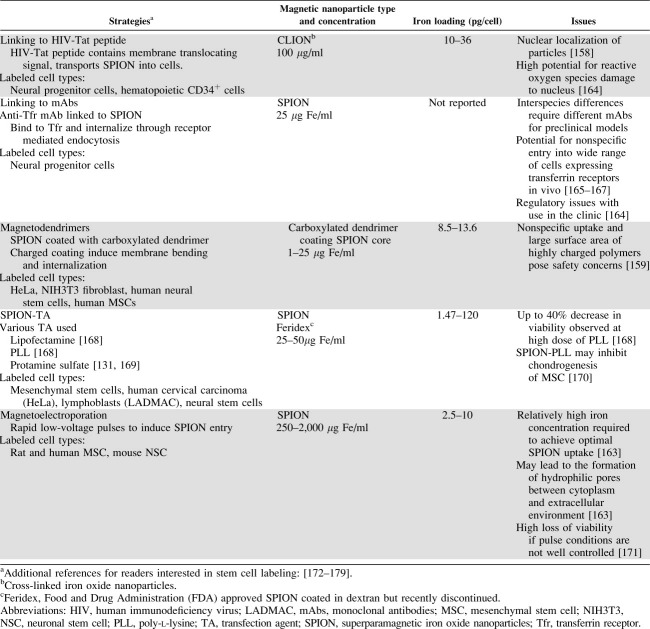

#### Use of Other Nanolabels Can Improve MRI Detection of SCs.

**Figure 3:**
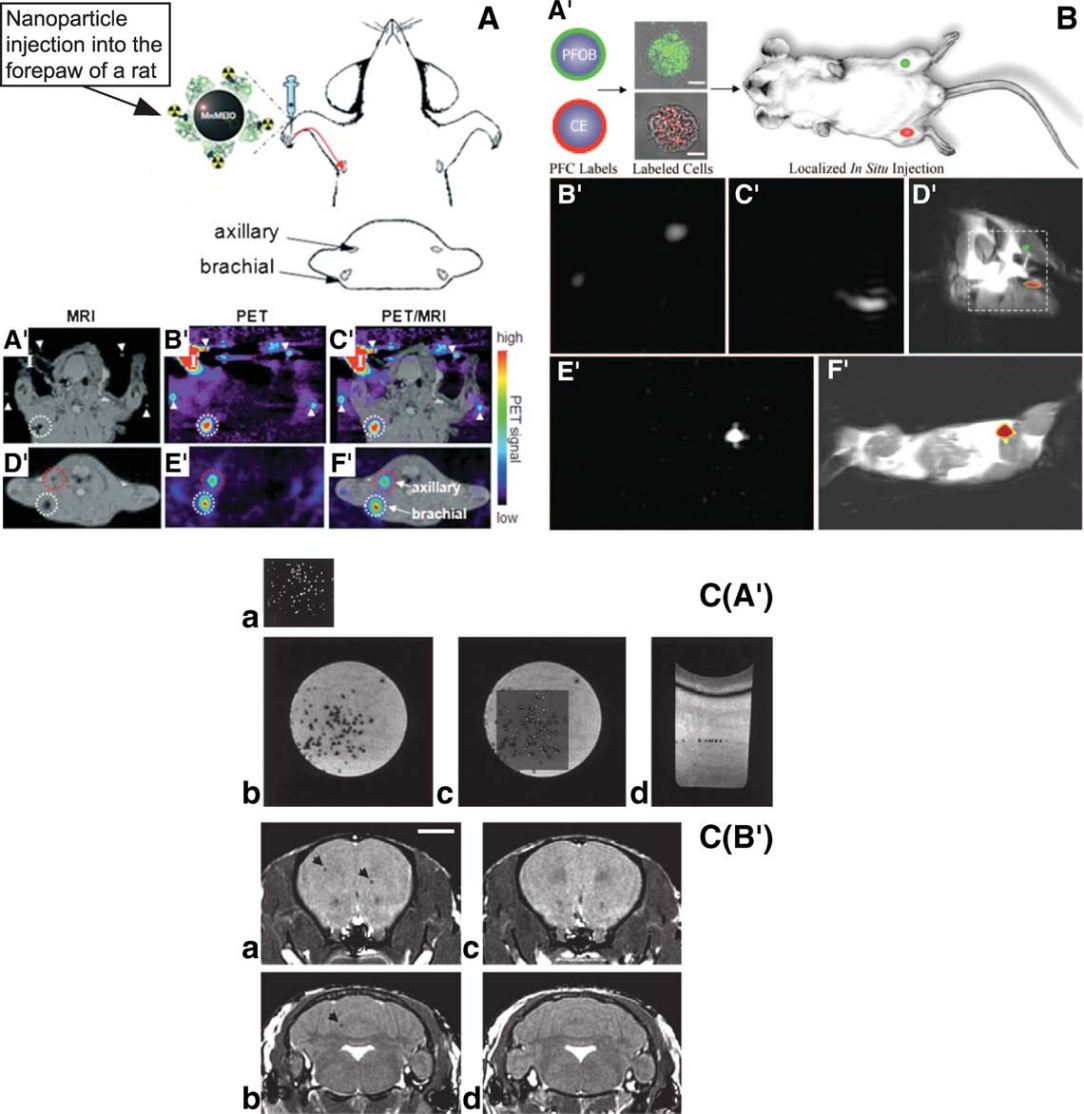
Approaches to improve stem cell tracking by MRI. **(A)**: PET-MRI dual modality imaging using multimodal nanoparticles: (magnetic nanoparticles + radionuclide, 124I), brachial (3 mm; A′, B′, C′) and axillary lymph nodes (D′, E′, F′) could be detected by superimposition (C′, F′) of anatomical MRI images (A′, D′) with the intense red signal images obtained with PET (B′, E′). Reproduced from [188], with permission from ©Wiley-VCH Verlag GmbH & Co. KGaA. **(B)**: 19F Rapid imaging of labeled mononuclear cells (human umbilical cord blood) at both research (11.7 Tesla) and clinical field (1.5 Tesla) strengths: Using multiple perfluorocarbon nanoparticles, green (PFOB) or red (CE), hot spot 19F images (B′: PFOB) and (C′: CE) were generated and could be superimposed with 1H MRI (11.7 T) for anatomical localizations to the mouse legs (D′). Similar results were obtained using 1.5 T MR (E′: 19F image and F′: superimposed 19F and 1H image). The authors were able to detect as few as 2,000 CE-labeled and 10,000 PFOB-labeled cells with 19F MR spectroscopy and 6,000 CE-labeled cells with 19F MRI in vitro. Reproduced from [187], with permission from *FEDN of AM Societies for Expreimental Bio (FASEB) Journal* via copyright clearance center, © 2006 by FASEB. **(C)**: Detection of Ultra Small Superparamagnetic Iron Oxide Nanoparticles (USPION)-labeled cells using fast imaging employing steady state acquisition pulse sequence on a 1.5 T clinical MRI scanner. (A′): Single USPION-labeled cells could be detected using a custom built gradient RF coil and optimized pulse technology. (a): fluorescent image of Dil/superparamagnetic iron-oxide nanoparticles (SPION)-labeled cells localized between two layers of gelatin in an ELISA well, (b) MR image, (c) fusion image, (d) Axial MR showing the localization of cells in a plane. Reprinted from [189], with permission from ©2003Wiley-Liss, Inc. a subsidiary of John Wiley & Sons, Inc. (B′): In vivo MR images detecting SPION-labeled macrophages (signal voids shown by arrows) injected into the mouse brain frontal cortex (a) and cerebellum (b). (c, d) represent the corresponding images of a control mouse. Reproduced from [190], with permission from ©2006 Wiley-Liss, Inc., a subsidiary of John Wiley & Sons, Inc. Abbreviations: CE, perfluoro15-crown-5 ether; MRI, magnetic resonance imaging; PET, positron emission tomography; PFC, Perfluorocarbon; PFOB, perfluorooctylbromide.

Given that signal gain (*T*1 contrast) is more specific and easier to interpret than signal loss (*T*2 contrast), paramagnetic manganese-oxide- or gadolinium-oxide-based nanoparticles (*T*1 contrast enhancers) may provide an attractive alternative [99,101,182]. Gd-oxide nanoparticles have appeal because Gd-chelates are approved for clinical MRI and have been used to trace human NSC or MSC [183]. However, potential mitochondrial toxicity [184,185] compounded by a requirement of greater molar quantities for optimal imaging has limited the interest in their use for MRI. Novel paramagnetic fluorinated nanoparticles have recently been shown to display high specificity with both clinical and high field MRI. Given the absence of endogenous fluorine (F) in the body, hot spot 19F MRI images of labeled cells were generated with negligible background. Ahrens et al., tracked 19F-nanoparticle-labeled dendritic cells (using cationic perfluoropolyether) to the regional lymph-node after injections into the foot pad of mice [186]. For a complete picture, though, the hot spot image requires overlaying with a simultaneous proton image (standard^1^H MRI) [150,187] (Fig. 3B). Despite the benefit of quantifying the labeled cells [191], sensitivity is low as the signal comes only from the labeled cells, while the proton signal draws from a much larger pool within the body and hence, is currently the preferred choice.

#### Other Approaches.

One approach is to utilize gene technology to introduce magnetic susceptibility enhancing genes [192], efficient transduction of these cells would be key to success of such an approach. Toward that end, the magnetic properties of virus or plasmid DNA-conjugated SPION have been harnessed through “magnetofection” to provide superior transduction (up to 500-fold increase) of “hard to transduce” cells with shorter incubation periods [193–195]. We have shown that Ad-conjugated SPION and magnetofection markedly improves the transduction of MSCs (low CAR expression) ex vivo while minimizing vector toxicity through a reduction in vector dose and incubation time (unpublished data).

Additionally, notable success of approaches employing modification of hardware and imaging protocols as well as synergizing different imaging modalities has pioneered new innovations for future research [105,114,196–202]. Some of these approaches are summarized in Table 4 and Figure 3.

**Table 4:** Approaches to improve detection of labeled stem cells by MRI

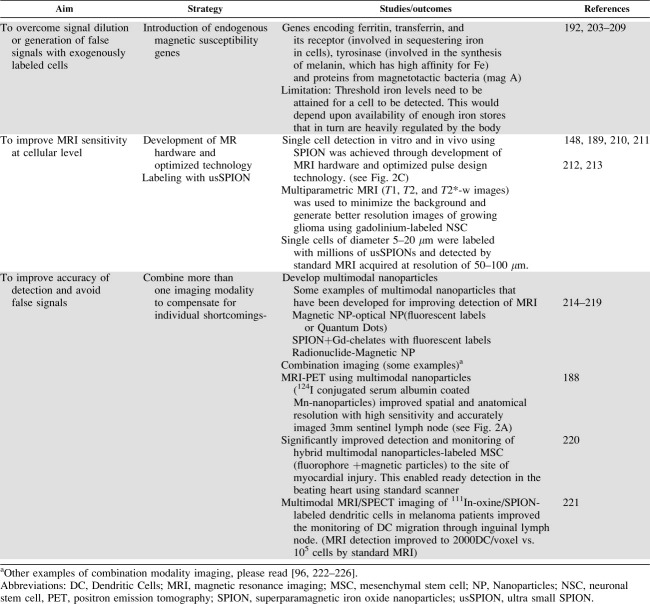

Thus, recent developments in SC, gene technology, and nanotechnology platforms against cancer have reached a junction where there is enormous potential to synergize their individual advantages to achieve concomitant tumor-targeted therapy and imaging.

## CONCLUSIONS AND PERSPECTIVES

The potential synergism between MSCs, gene-therapy, and magnetic nanoparticles offers an exciting innovation that may offer cancer patients greater treatment and disease management options and ultimately better quality of life. The advances in nanotechnology may be combined with MSCs to facilitate their tracking and provide accurate details about their location, viability and survival. For effective cellular therapy of cancer, the carriers need to target cancer deposits irrespective of their size and location, should be traceable and should survive long enough to deliver the therapeutic payload. This will require real-time imaging ability with high spatial and temporal resolution as well as stringent target specificity.

Current clinical probes generally cater to a single imaging modality, however, it is now clear that combining the attributes of multiple modalities will be required to provide a comprehensive assessment of events as they occur [114]. Indeed, this concept, now explored by various research groups will soon be a preferred choice for clinical application. Again, the flexibility of nanotechnology platforms may be a great ally in imaging cell-based therapies. For example, the imaging potential of MSCs labeled with magnetic nanoparticles conjugated with radionuclides will allow the combined advantages of short-term PET sensitivity and the long-term signal persistence of MRI. Further, development of multimodal smart nanoparticles that can simultaneously image and treat cancer with real time monitoring of associated events [114,188] is now feasible through the versatility of nanotechnology. However, these particles need to be exhaustively assessed for their biocompatibility and intracellular or in vivo toxicity before they achieve widespread applicability in the clinic.

Given a relatively poor understanding and ability to control MSCs in vivo behavior, their application as carriers of contrast agents may not be safe [227]. This may be resolved to some extent by combining imaging with a backup suicide gene technology to eradicate misbehaving cells. This can be achieved through smart combinations with tools of gene therapy, for example, through introduction of suicide genes with regulatable promoters. For example, the use of Herpes Simplex Virus (HSV)/tk Gene Directed Enzyme Prodrug Therapy (GDEPT) and radioactive substrate (^18^F-9-(4-[^18^F]Fluoro-3-Hydroxymethylbutyl) Guanine (18F-FHBG)) has been successful in both human and animal studies for PET imaging [228]; the presence of HSV/tk suicide gene can serve as an additional control to eliminate the transduced cell by treatment with the prodrug (Ganciclovir and Acyclovir) which is then converted to a toxic drug by tk. Thus, magnetically labeled MSCs with HSV/tk GDEPT would allow MRI-PET along with the control of cell survival as needed. Both nanoparticles and MSCs can carry gene vectors, hence, there is scope for endogenous expression of reporter genes under tissue or lineage specific promoters [229] or expression of magnetic susceptibility enhancing genes to enhance the accuracy of the imaging data. Such adjuncts may allow additional assessment of cell viability, survival and fate. Use of reporter genes green fluorescent protein or luciferase [192] regulated by lineage-specific promoters may help detect SCs following differentiation, for example, use of cardiac-specific α myosin heavy chain promoter to detect SC conversion to cardiac myocytes [230,231], Tyrosine kinase with immunoglobulin-like and EGF-like domains 1 (TIE) promoter [229] to detect endothelial differentiation, and osteopontin or osteocalcin promoters to detect osteogenic changes [232].

Taking into account the limitations and attributes of different types of SCs, MSCs offer a feasible option in clinical oncology. New sources of MSCs are under development, including those from adipose tissue [30,76,77] and umbilical cord blood [233,234] and show similar tumor homing and functional capacity as BM-derived MSCs. The wide availability of MSCs from these sources and development of well-characterized immortalized clonal stem cell lines may also ease the practical application of MSCs in the clinic. In particular, the potential for MSCs to be transplanted across MHC barriers in humans [71] can be further explored to facilitate ease of donation in future clinical contexts. It must be noted that the specificity of this system is highly conditional on the tumor homing abilities of MSCs and their in vivo behavior, making this a priority research area. Enhanced insight into the mediators of homing will allow for active targeting of tumors by inducing MSCs to overexpress target receptors for homing. Also, with increasing knowledge of mechanisms or pathways involved in SCs migration, efforts are being directed toward developing specific ligands to target lesions, for example, to direct cells to CXCR4/SDF1 axis to facilitate MSCs tumor tropism [59]. Recent evidence of increased MSCs engraftment in tumors following irradiation (releases chemotactic signals) [235,236] also indicates the potential of using MSC-based gene therapy as an adjuvant following radiotherapy to maximize the removal of residual disease. Finally, highly specific delivery and individualized therapy may be achieved by the choice of therapeutic genes and manipulation of the vectors depending on the cancer type and degree of aggressive therapy required.

Overall, it is clear that there is no single magic bullet to overcome the complexity and heterogeneity of cancer. Multifaceted approaches that exploit the best attributes of MSC biology, nanotechnology, gene-technology, and gene therapy have the potential to overcome hurdles encountered when each is used alone. However, the possibility that such multidimensional modifications may also enhance the danger of unwanted changes in MSCs functional phenotype such as gain of tumorigenic potential or loss of specific migration, cannot be ignored. A rigorous characterization of modified cells with focus toward addressing the potential regulatory issues would be crucial to achieve their speedy translation to the clinic.

## Disclosure of Potential Conflicts of Interest

The authors indicate no potential conflicts of interest.
